# Molecular Mechanisms and Anticancer Therapeutic Strategies in Vasculogenic Mimicry

**DOI:** 10.7150/jca.34171

**Published:** 2019-10-18

**Authors:** Xue Zhang, Jigang Zhang, Heming Zhou, Guorong Fan, Qin Li

**Affiliations:** Department of Clinical Pharmacy, Shanghai General Hospital, Shanghai Jiao Tong University School of medicine, No.100 Haining Road, Shanghai, 200080, P.R. China.

**Keywords:** vasculogenic mimicry, molecular mechanisms, miRNAs, lncRNAs, circRNAs

## Abstract

Vasculogenic mimicry (VM) is a vascular formation mechanism used by aggressive tumor cells. VM provides an alternative pathway for adequate blood perfusion and challenges the traditional angiogenesis mechanism that depends only on endothelial cells (ECs), as VM-forming tumor cells express a mixed endothelial/tumor phenotype. VM is closely correlated with tumor invasion, migration, and progression. Hence, anticancer therapeutic strategies targeting VM biogenesis are essential. It is widely acknowledged that the VM formation mechanism involves multiple pathways. The purpose of this review is to describe the potential molecular mechanisms related to different pathways and discuss the involvement of microRNAs (miRNAs), long non-coding RNAs (lncRNAs), and circular RNAs (circRNAs) in VM formation. Moreover, we discuss the significance of VM in clinical practice and present new anticancer therapeutic strategies that target VM.

## Introduction

The tumor formation mechanisms are complex and diverse. Tumors characterized by a rapid growth speed have unfavorable prognosis, and high mortality, as they are difficult to diagnose early and there are no effective measures to treat them. Angiogenesis is one of the mechanisms through which tumor cells have an adequate blood supply and nutrition for their growth. However, many conventional antiangiogenic drugs adopted clinically produce disappointing results. The discovery of vasculogenic mimicry (VM) provides a new therapeutic opportunity for patients battling aggressive tumors for which treatment with conventional antiangiogenic agents is limited. Since VM was first discovered, it has been consistently found in different cancer types, including hepatocellular carcinoma (HCC) 1, breast carcinomas 2, ovarian carcinoma 3, lung cancer 4, glioma 5, and renal carcinoma 6. Despite the high amount of literature on VM, the biogenesis mechanisms of VM are still not fully elucidated, and further research on VM base biology is critically important. The purpose of this review is to discuss the potential molecular mechanisms hypothesized for VM, and the involvement of miRNAs, lncRNAs and circRNAs as well as discuss the clinical significance of VM and report new anticancer drugs that target VM.

### Conceptual progress on VM biology

**Definition of Vasculogenic mimicry.** VM is the *de novo* formation of a perfused, matrix-rich, vasculogenic-like network of blood vessels by aggressive tumor cells. VM mimics the embryonic vascular network pattern to provide sufficient blood supply for the growth of the tumor. The initial morphologic and molecular characterization of VM was by the Maniotis group, which revealed that human melanoma cells formed channels, networks, and tubular structures that are rich in laminin, collagens IV and VI, and heparin sulfate proteoglycans. The newly formed network contained plasma and red blood cells to facilitate tumor perfusion, remold the extracellular matrix, and change the cell phenotype 7.

**Plasticity and perfusion ability of VM.** Cancer cells capable of VM present multipotent, stem cell-like phenotypes, including both a tumor and endothelial phenotype, indicating a remarkable degree of plasticity. A seminal example of VM functional plasticity was the transplantation of fluorescently labeled metastatic melanoma cells into a surgically induced ischemic microenvironment in the hind limbs of nude mice, which demonstrated the powerful influence of the tumor microenvironment on the transendothelial differentiation of aggressive melanoma cells and provided a new perspective on tumor cell plasticity 8. A previous study investigated the plasticity of tumor cells in melanoma VM, reporting that the hypoxic microenvironment in metastases promotes to a phenotype switch that allows melanoma cells to physically contribute to the blood vessel formation 9. A recent study revealed that the Epstein-Barr virus (EBV) induced tumor cell plasticity by promoting VM formation 10. VM facilitates perfusion in rapidly growing tumors by transferring fluid from leaky vessels and/or by linking the VM network with the endothelial-lined vasculature. This was demonstrated by Doppler imaging of microbeads circulation, showing physiologic perfusion of blood between mouse endothelial-lined neovasculature and VM networks in human melanoma xenografts 11.

**Types of VM.** In aggressive malignant tumors, two distinctive VM patterns have been identified: matrix VM and tubular VM. Matrix VM is composed of a basement membrane that is surrounded by tumor cells rich in fibronectin, collagens, and laminin. The presence of matrix VM is an unfavorable prognostic factor compared to tubular VM in HCC patients 12. Tubular VM is composed of tumor cells that mimic the normal endothelium to form perfused channels. However, in many tumors, it is common to have both angiogenic and non-angiogenic areas. Interestingly, in the absence of angiogenesis and normal blood vessels exploitation, VM can act in a non-angiogenic way to provide oxygen and nutrients to the tumor 13.

**Figure 1:**
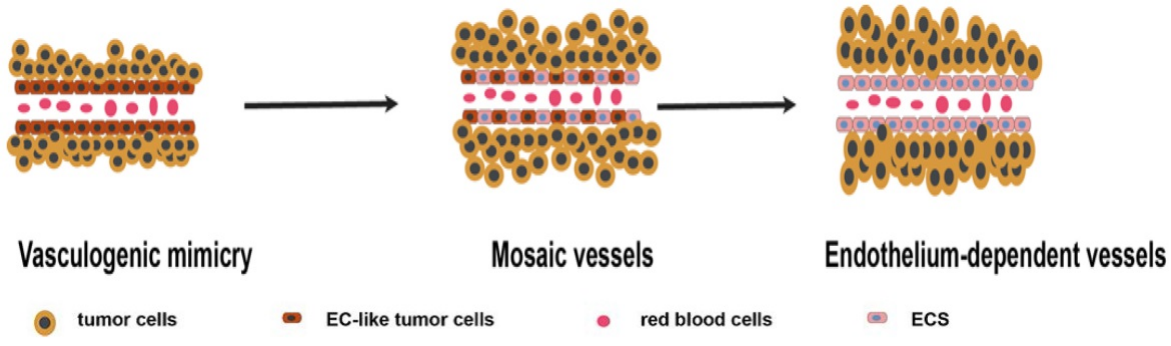
Schematic illustration showing the three microcirculation patterns associated with VM. In the early stages, VM play a major role in providing blood supply. With the increase of tumor size, tumor cells lining the wall of VM vessels are replaced by endothelium. MVs is the transitional state between EVs and VM. Finally, EVs become the major pattern of blood supply.

**Microcirculation patterns associated with VM.** Different studies have proposed three microcirculation patterns: VM, mosaic vessels (MVs), and endothelium-dependent vessels (EVs), representing different stages of tumor growth. In the early stages, VM plays a major role in providing blood supply. With the increase in tumor size, tumor cells lining the wall of VM vessels are replaced by endothelium cells. At this point, MVs represent a transitional state between EVs and VM. Finally, EVs become the major blood supply pattern 14 (Figure 1). A recent research showed that VM acts as a part of the functional microcirculation, cancer cells within the tumor-lined vascular channels can easily transfer into endothelial-lined blood vessels in VM angiogenesis junction, consequently, contributing to tumor invasion and metastasis 15.

**VM assessment.** A positive staining pattern with Periodic Acid-Schiff stain (PAS) along with the absence of CD31 or CD34, two classical markers, indicates the existence of matrix-associated vascular channels. Thus, VM can be diagnosed by performing immunohistochemical analysis (IHC) in tumor samples. VM positive samples have a positive PAS staining pattern and a negative CD31 staining pattern 7. Interestingly, a recent study found that VM channels also exist in CD31/CD34-positive gastric adenocarcinoma cells, probably because the genetically deregulated tumor cells express angiogenic and vasculogenic markers 16.

### Potential molecular mechanisms involved in VM

**Relationship among EMT, CSCs, and VM**. The mechanism of VM biogenesis is closely related to the epithelial-to-mesenchymal transition (EMT) and to cancer stem cells (CSCs).

**Figure 2:**
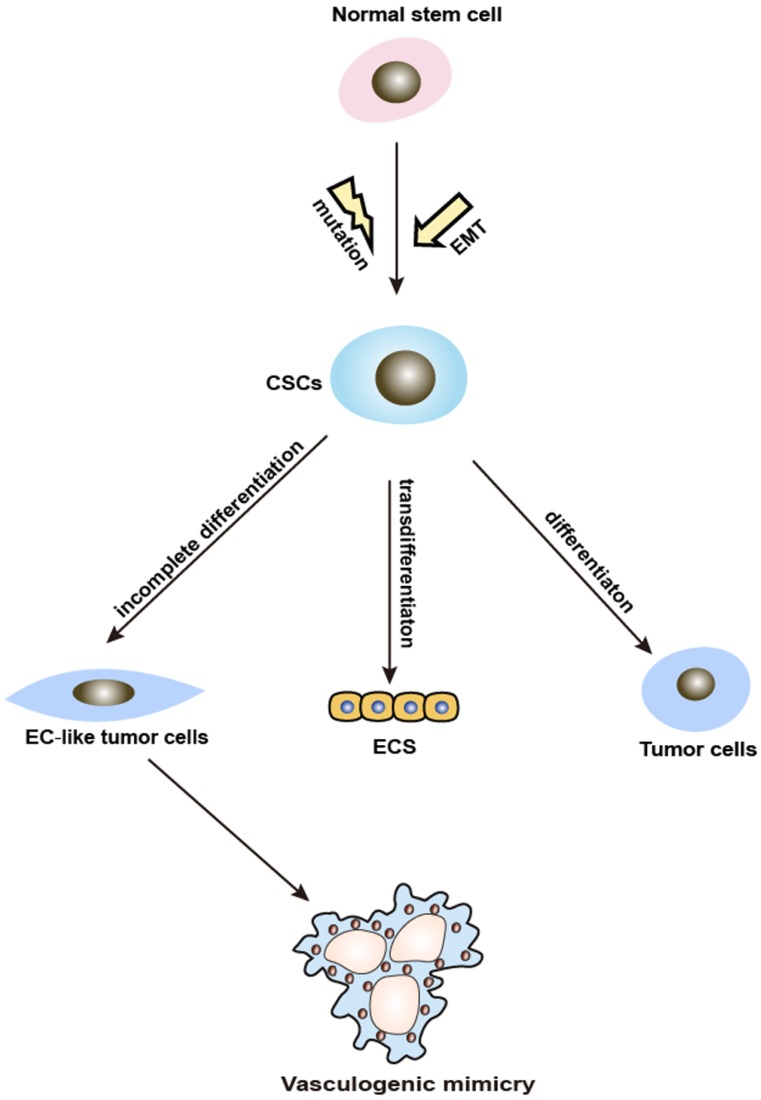
Schematic illustration showing the involvement of CSCs and EMT in VM formation. VM: vasculogenic mimicry; CSCs: cancer stem cells; EMT: epithelial-mesenchymal transition.

**CSC and VM.** CSCs are a small proportion of tumor possessing reversible self-renewal capabilities, which can differentiate into multiple cell types. It is widely accepted that normal stem cells differentiate into CSCs through a mutation process. However, recent findings indicate that CSCs could have originated and be maintained through EMT 17. The increased knowledge about CSCs revealed a close connection to VM. It has been found that CSCs stimulate VM in the tumorigenic microenvironment by differentiating or transdifferentiating tumor and ECs, lining up to form branching tubes and lumens resembling a vascular network which provides nutrition for the tumor mass. Ultimately, the tubes extend, merge, and start transferring blood cells 18 (Figure 2). It has also been demonstrated that because of the loss of stem cell capacity, VM gradually skews toward a vascular phenotype 19. Additionally, a handful of studies suggested that CSCs markers are also associated with VM. VM-forming cells contributing to the tumorigenicity are characterized by the expression of the CSCs markers CD133 and aldehyde dehydrogenase 1 (ALDH1) 20. Liu et.al revealed that USP44+ CSCs subclones with an ALDH1+/USP44+/IL6+/IL8+ phenotype may promote VM and tumor aggressiveness 21. In conclusion, CSCs can induce VM formation in two ways: incomplete differentiation and the up-regulation of CSCs-associated molecules. Further investigations of CSCs markers to target VM and the link between CSCs and VM may contribute to the discovery of new anticancer treatments to prevent VM formation.

**EMT and VM.** The involvement of EMT in cancer is currently widely accepted. EMT is a biological process that plays a central physiological role during embryogenesis and a pathological role in cancer progression. The activation of EMT triggers tumor cell invasion and the formation of metastasis in distant organs. The high expression of EMT-associated adhesion molecules can contribute to the VM-forming process. The molecular mechanisms of ZEB1-induced VM formation were confirmed to be dependent on mediation of the Src signaling pathway, which also plays an important role in EMT as well as in maintaining CSCs properties 22. Notably, Langer et.al demonstrated that miRNA clusters repressed by ZEB1 stimulated VM through autocrine signaling in breast cancer cells 23. Further studies showed that EMT regulators such as Twist, Snail, and Slug were closely related to VM 24, 25. Meng et.al found that Twist1 interacts with Hsp90β, activating VE-cadherin transcription to induce EMT and promote VM in HCC 26.

Both EMT and CSCs confer resistance to chemotherapy and are supposed to be the underlying causes of low survival rates in patients with aggressive tumors. Therefore, given the relationship between EMT, CSCs, and VM, it is plausible to hypothesize therapeutic strategies targeting EMT and CSCs as a promising candidate for VM-related therapies.

**Tumor Microenvironment.** The extracellular microenvironment (ECM) is an important structural element for tumor cells. ECM can be changed by cellular processes and in turn can exert an influence on cellular activities. This mutual interaction plays an integral role in VM. ECM composition regulates the acquisition of the VM phenotype in CSCs. In the early tumor ECM, CSCs can secrete a high amount of angiopoietin factors such as IGFBP1/2/3, MCP1, IL8, EGF, and VEGF, to stimulate CSCs growth/self-renewal and start the VM process 27. Distinct collagen architectures in the ECM affect tumor cell motility behaviors linked to VM, and various ECM molecules, such as COL4A1, JAG1, and THBS1, may facilitate the emergence of VM 28. A study reported that highly aggressive melanoma cells can alter their ECM to form VM tubular networks and that the cooperative interaction of Matrix metalloproteinase-2 (MMP-2) -14 or Ln5γ2 chains is required for VM formation 29.

This discovery opened up a new avenue of research on the role of other MMPs in VM formation. It has been found that, in renal carcinoma cells, downregulation of MMP-9 leads to the decrease of VM formation, revealing that MMP-9 is necessary for VM formation in this cell type 6. Another study demonstrated that, in melanoma cells, Myoferlin has an effect on VM formation by mediating the expression of MMP-2 and inducing EMT 30. A further report showed that MMP-2 activates the epidermal growth factor receptor (EGFR), enhances cytoskeletal rearrangement and facilitates VM formation. However, MMP-13 lowers the EGFR/F-actin expression, degrades ECM components and hinders VM formation. Although both MMP-2 and MMP-13 promote the activity of Ln-5 cleavage and degrade ECM components, they exert distinct influences on large cell lung cancer cells 31. Further research is needed to better understand the role of the distinct MMPs in VM formation. MMP-2, MMP-13, and MMP-9 could be used as therapeutic targets to inhibit VM in anti-tumor therapy.

**VE-cadherin.** VM-forming tumor cells can express both endothelial and tumor phenotypes. Vascular endothelial-cadherin (VE-cadherin), a calcium-dependent protein, is the key factor regulating cell-cell adhesion in ECs, and it is the most important molecular determinant for the acquisition of VM capabilities 32. A previous study attempted to elucidate the molecular mechanism of VE-cadherin involvement in VM, showing that the downregulation of VE-cadherin impaired the ability to form VM and the plasticity of aggressive human melanoma. They also showed that VE-cadherin is often overexpressed in highly aggressive tumor cells compared with non-aggressive ones 33. Interestingly, VE-cadherin could co-localize with phosphorylated Eph receptor tyrosine kinase A2 (EphA2), which is an important factor promoting the formation of a vessel-like network 34. A recent study reported that VE-cadherin-positive small cell lung cancer (SCLC) cells were able to show VM and were more resistant to cisplatin than VE-cadherin negative cells 4. Another study revealed that in melanoma cells the activation of focal adhesion kinase (FAK) increased the expression of VE-cadherin and was positively correlated to VM formation 35.

**EphA2.** The main role of VE-cadherin in VM is to mediate EphA2, an epithelial cell-associated kinase that is phosphorylated when bound to its ligand, ephrin-A1 36. Knockdown of VE-cadherin reduced the phosphorylation of EphA2 on the cell surface, while down-regulation of EphA2 expression had no effect on VE-cadherin, suggesting that EphA2 may be a downstream regulator of VE-cadherin expression 37. Furthermore, it has been demonstrated *in vivo* that the reduction of EphA2 expression significantly inhibits VM formation and suppresses the invasion, proliferation and clonogenicity capabilities of melanoma tumor cells 38. Additionally, a previous study showed that EphA2 may be an EMT mediator, contributing to VM formation in head and neck squamous cell carcinoma 39. Current report revealed that serum activated EphA2 and up-regulated Twist/VE-cadherin, which in turn activated AKT that up-regulated MMP-2 and LAMC2, thereby inducing the invasion and VM of PC-3 human prostate cancer cells 40.

**PI3K.** Phosphoinositide 3-Kinase (PI3K) is a family of intermediate signaling molecules that are involved in various cell responses, particularly in the signal transmission from the cell surface to the cytoplasm pathway. PI3K proteins are commonly found in a wide variety of cancers and have been recognized as a diagnostic marker of cancer 41. High PI3K-mediated phosphorylation levels of EphA2 and VE-cadherin increase MMP-14 and MMP-2 activity, which in turn promotes the cleavage of the Ln5γ2-chain into γ2′ and γ2 fragments, ultimately leading to VM formation 42, 43. It has been shown that AKT, also known as protein kinase B, is a downstream effector of PI3K proteins and is a critical vasculogenesis regulator 44. In HCC, the PI3K/AKT signaling also regulate the MMP-9 levels and activity, contributing to ECM remodeling towards VM 45. Hence, inhibiting the PI3K/AKT pathway may provide a new target for anti-VM therapy.

**ERK1/2.** Similar to the PI3K-mediated signaling pathway, the extracellular signal-regulated kinase (ERK) is another key signaling pathway involved in the cell signal transduction process, participating in various physiological and pathological cellular processes, such as tumor cell invasion, proliferation, migration, and apoptosis 46. It has been showed that when human hepatoma cells are exposed to hypoxic conditions, the activation of ERK1/2 mediated by mitogen extracellular kinase (MEK) promotes the expression of VE-cadherin, ultimately contributing to VM formation 1.

**FAK.** FAK is a key molecule in the process of VM formation, through its interaction with PI3K proteins 47. Additionally, FAK is a downstream effector of EphA2 and plays a critical role in highly aggressive GBC-SD cell growth. It has been showed that upregulation of the EphA2/FAK/Paxillin signaling pathway promoted VM formation 48. FAK is also a pivotal mediator of the aggressive melanoma phenotype, which is characterized by an increased expression of ERK1/2 to regulate the levels of urokinase activity or to enhance the expression of MMP-2 and MMP-14 activity, both signaling pathways contribute to VM formation 49. Another recent study reported that in NSCLC, the FAK/AKT signaling pathway is involved in the cyclin-dependent kinase 5-mediated VM formation 50.

**VEGF-A/VEGFR.** Vascular endothelial growth factor-A (VEGF-A) is a major regulator of vascularization and is critical for vascular EC proliferation, migration, and survival, especially when it is bound to vascular endothelial growth factor receptor 1 (VEGFR1). Activation of the PI3K/AKT pathway by VEGFR1 is involved in endothelial angiogenesis, whereas activation of the Src and ERK1/2 pathways results in tumor cell invasion and proliferation 51. In addition, in melanoma cells, the integrin-mediated signaling pathway involving VEGF-A/VEGFR1/PI3K/PKCα, is required for VM formation 52. Moreover, it has been established that in ovarian carcinoma cells, VEGF-A takes part in the formation of VM via indirectly upregulating the expression of EphA2, MMP-2, MMP-9, and VE-cadherin 3. The increased expression of VEGFR-2, another type of vascular endothelial growth factor receptor, in CSCs-derived tumors, influences the formation of VM networks 27. A recent study reported that in glioma stem cells, autophagy-induced phosphorylation of the kinase insert domain receptor of VEGFR-2 contributes to VM formation 53. Other studies supported the evidence that the Hippo pathway is a key regulator of VM and angiogenesis through the VEGF-Induced PI3K/MAPK signaling 54. VEGF binding to semaphorin4D (SEMA4D) had a synergistic effect on VM formation 55. However, the role of VEGF signaling in mediating VM is controversial. A recent study reported that VEGF-A silencing upregulated the expression of MMP-2 and VM marker VE-cadherin, leading to VM formation. Thus, VEGF-A inhibition may have a dual biological role that could confound its clinical effectiveness 56.

**PEDF.** Various evidences suggest a role for pigment epithelium-derived factor (PEDF), a serpin protease inhibitor, in suppressing angiogenesis by inhibiting VEGF-induced phosphorylation of VEGFR-1 57. Furthermore, a study showed that PEDF silencing enhances the capability of forming VM in poorly aggressive melanoma cells lines, implying that the expression of PEDF is negatively associated with VM formation 58.

**TF, TFPI-1, and TFPI-2.** Tissue factor (TF), TF pathway inhibitor 1 (TFPI-1), and TFPI-2 are overexpressed in aggressive melanoma cells. All these three genes play a critical role in mediating the coagulation pathway. TFPI-1 was shown to regulate the anticoagulant function of TF, which is associated with perfusion of VM, whereas TFPI-2 seems to contribute to VM plasticity through increasing the MMP-2 activity and influencing the extracellular matrix remodeling 11.

**Nodal.** Nodal is a member of the superfamily of the transforming growth factor β (TGF-β) family. It plays a critical role as embryonic morphogen. Nodal regulates tumor cell plasticity, the transendothelial phenotype, and VM formation by binding to cripto-1, ALK4/5/7 and type 2 (ACTR-IIB) proteins to phosphorylate SMAD2/3, which in turn translocates to the nucleus where it mediates gene expression 59, 60. *In situ* hybridization found that the expression of Nodal mRNA is consistent with the formation of vasculogenic networks and that the downregulation of Nodal contributes to the reduction of VE-cadherin, thereby influencing VM formation 61. Recently, it was found that in MCF-7 cells, Nodal, through the Smad2/3 pathway, regulates the transcription factors Snail and Slug and increases MMPs expression, thereby inducing EMT and VM formation 25.

**Notch.** Similar to Nodal, Notch is essential for embryonic development. It is well known that four transmembrane Notch receptors (Notch1, 2, 3, 4), coupled with five ligands, participate in the vertebrate embryogenesis process. Emerging evidence showed molecular cross-talk between Nodal and Notch. Nodal signaling is initiated through a series of proteolytic cleavages that release the Notch intracellular domain (NICD). Then, NICD translocates to the nucleus, activating the transcription of Nodal 62. Consistent with this, the co-expression of Nodal and Notch4 is required for tumor cell proliferation and survival. A study showed that the inhibition of Notch4 reduces the expression of VE-cadherin and blocks VM in a Nodal-dependent manner, implying that the Notch4-N odal signaling axis may be a key mediator of vasculogenic networks 63. In addition, a recent study found that in HCC, Notch1 expression is associated with VM formation by mediating the EMT pathway, while in gastric cancer by increasing VEGF secretion 64, 65. Furthermore, Notch3 silencing using lentiviral shRNA attenuated both tumor growth and VM in melanoma stem-like cells, suggesting that Notch3 is closely associated with tumor angiogenesis 66.

**TGF-β.** TGF-β superfamily plays essential roles in cell growth, apoptosis, motility, and invasion. Various studies showed that members of the TGF-β superfamily have both negative and positive effects on carcinoma cells. The relationship between various TGF-β proteins and VM is well established. Experimental evidence in aggressive tumor cells indicates that the binding between Endoglin (ENG) and TGF-β leads to neoangiogenesis and VM 67. TGF-β inhibition in U251MG cells can also reduce the expression of MMP-14, resulting in a significant decrease in VM formation 5. Furthermore, blocking TGF-β signaling by silencing TGF-ΒR1 in HCC cells attenuates VE-cadherin/MMP-2/LAMC2 expression and inhibits cancer-associated fibroblast (CM-CAF)-promoted VM formation 68. Thus, TGF-β-related signaling pathways could be potential targets for anti-VM cancer therapy.

**Hypoxia.** A large body of evidence supports the role of hypoxia in maintaining the stem cell-like phenotype of tumor cells and in promoting tumor invasion, metastasis, and VM. In melanoma, HCC, glioblastoma, and breast cancer, hypoxia is capable of inducing VM channel formation 7, 69-71. Recently, the relationship between hypoxia and VEGFA has been further investigated. VEGFA is a critical downstream effector in hypoxia-induced VM in human salivary adenoid cystic carcinoma (SACC) tissues. This process is mediated by EMT and CSC 72. Moreover, hypoxia-inducible factor-1 (HIF-1) is involved in VM formation either by directly regulating VEGF-A, VEGFR1, EphA2, Twist, Nodal, and COX2 expression or by indirectly regulating VE-cadherin and TF expression. The hypoxia-induced regulation of Nodal expression occurs via a combinatorial mechanism mediated by HIF-1α and stabilized by the Notch protein NICD, which activates the Notch signaling pathway 73, 74. Another study supported the relationship between HIF-1α, EMT, and VM. They showed that, under hypoxic conditions, HIF-1α affects VM formation by mediating EMT in HCT-116 75. Additionally, it has been shown that HIF-1α targets LOXL2, which in turn mediates VE-cadherin, E-cadherin, and vimentin expression, thus contributing to EMT and VM formation 76. Both HIF and Twist are transcription factors with similar functions, in the VM formation process. Other than HIF-1α, a recent report showed that in pancreatic cancer cells, also HIF-2α can interact with Twist1 mediating the VM process 77. Furthermore, a strong positive correlation was demonstrated in glioblastoma cells between hypoxia-induced VM, macrophage migration inhibitory factor (MIF) and C-X-C motif chemokine receptor 4 (CXCR4) co-localization, and HIF-1α levels 70.

**Twist1/2.** Twist1/2, two transcription factors who play a key role in EMT, are correlated with angiogenesis and VM formation. Upregulation of Twist1 in HCC cells enhances the expression of VE-cadherin and MMPs, which is ultimately critical to VM formation. Moreover, the EMT marker E-cadherin is suppressed following the upregulation of Twist1, suggesting a connection between Twist1, EMT, and VM 24. Furthermore, Bcl-2 can enhance the expression of Twist-1 to promote VM formation through EMT 78. Likewise, the HMGA2-regulated Twist-1/VE-cadherin pathway enhances the expression of MMP-2, thereby inducing VM 79. Additionally, a recent study found that in HCC cells Protease-activated receptor-1 (PAR1) increases the Twist1 transcription activity both *in vitro* and *in vivo*, thereby promoting epithelial-endothelial transition (EET) and facilitating VM formation 80.

**COX-2.** Cyclooxygenases-2 (COX-2) is a key enzyme in prostaglandin E2 (PGE2) synthesis and has been found to increase tumor-associated VEGF expression through the protein kinase C (PKC)-mediated pathway in non-small cell lung cancer 81. PEG2 binding to Prostanoid receptors (EP1, -2, -3, -4) activates EGF receptor (EGFR) signaling and the PKC-mediated ERK1/2 pathway, which promotes tumor cell invasion, metastasis, and proliferation 82. Another study showed that in breast cancer cells, the overexpression of COX-2 promoted the formation of vascular channels, whereas low levels of COX-2 did not, implying that COX-2 is essential for VM. Furthermore, the COX-2/PEG2/EP3 signaling pathway regulates the expression of MMP-2 to form vasculogenic structures 83, 84. Interestingly, a recent study found that both M_2_ macrophages and the PEG2/EP1/PKC signal transduction pathway participated in the process of VM formation by activating COX-2 85.

**Figure 3:**
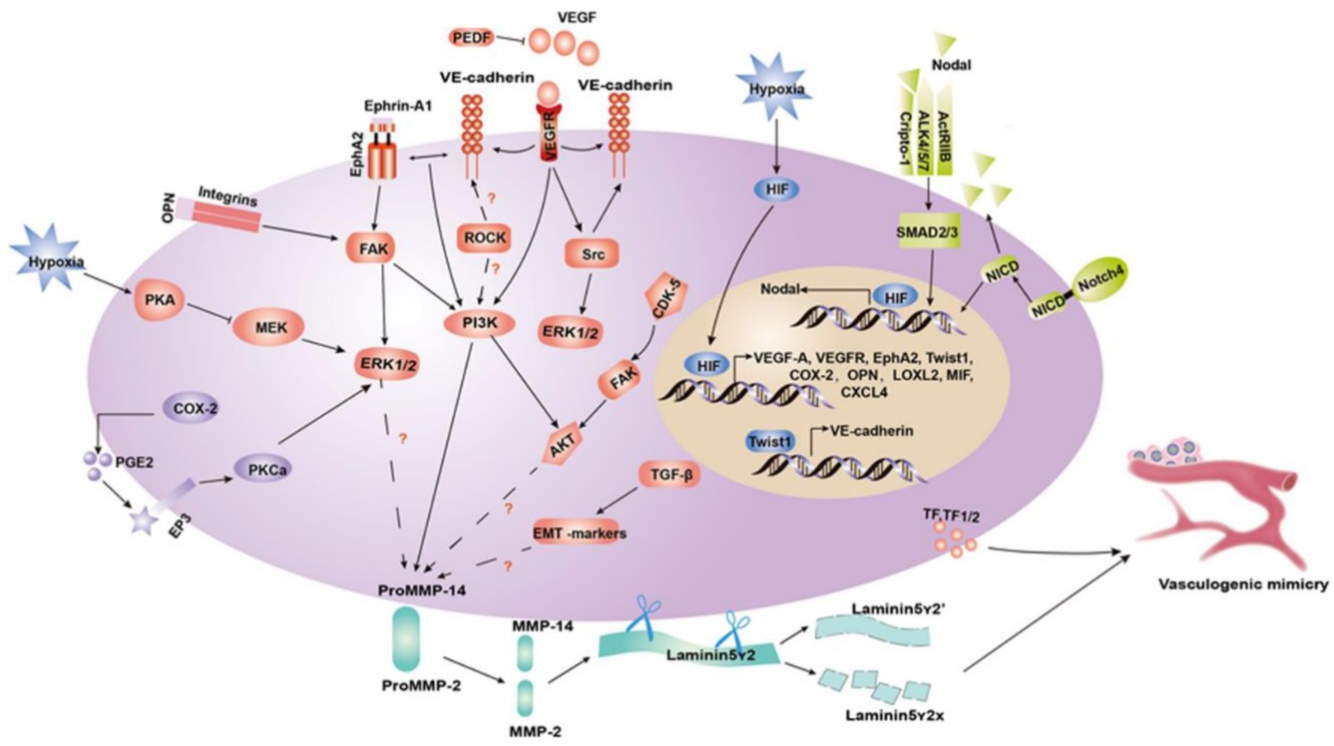
** Schematic model of a molecular mechanisms implicated in tumor cell VM. (1**) Tumor microenvironment components, including MMP-2 and MMP-14, facilitate the cleavage of Ln5γ2 into γ2 and γ2' fragments to contribute to ECM remodeling in VM. **(2)** PI3K, ERK1/2 and AKT are in intermediate signaling pathways that can influence the tumor microenvironment and take part in the process of VM formation through the VE-cadherin/EphA2/FAK/ERK1/2, VEGF-A/VEGFR1/PI3K/PKCα, COX-2/PEG2/EP3/PKCα/ERK1/2, and CDK5/FAK/AKT axis. **(3)** Nodal regulates VM formation by binding to cripto-1, ALK4/5/7 and ACTR-IIB, and then phosphorylating SMAD2/3, which translocates to the nucleus where it mediates gene expression. **(4)** HIF-1 induced by hypoxia directly regulates the gene expression of VEGF-A, VEGFR, EphA2, Twist, COX-2, OPN, LOXL2, MIF, and CXCL4. The hypoxia-induced regulation of Nodal expression occurs via a combinatorial mechanism that mediates HIF-1α and stabilizes the Notch protein NICD, activating Notch signaling.

**RhoA/ROCKs.** The Rho kinases (ROCKs) family, which includes the two isoforms ROCK1 and ROCK2, are serine/threonine kinases acting downstream of the Rho GTPases (RhoA, RhoB and RhoC). ROCKs play a major role in regulating actin dynamics such as actin-myosin-mediated contractile processes, by phosphorylating the myosin light chain (MLC) and LIMK1/2. This process influences cell adhesion, cell motility, and invasiveness 86. A growing number of studies reported a correlation between Rho GTPases and VM-associated markers such as VE-cadherin and MMPs in various cancer cells, implying that Rho GTPases are involved in the VM formation process 87, 88. In our previous study, blocking the ROCKs pathway in MHCC97H cells inhibits the expression of VM-related factors such as, EphA2, VE-cadherin, PI3K, MMPs and Ln5γ2. Furthermore, we suggested that ROCKs, rather than RhoA, participate in the formation of VM channels 89. In the subsequent study, we demonstrated that the activated RhoC/ROCK2 promotes VE-cadherin and MMP-2 expression, increasing the EMT occurrence by upregulating the ERK/MMPs signaling, and ultimately promoting VM formation. Furthermore, our results showed for the first time that RhoC/FAK/paxillin is involved in VM formation 90. Moreover, another recent study showed that in NSCLC cells, Sema4D activates the RhoA/ROCK pathway to regulate tumor cell plasticity, migration, and VM formation (Figure 3) 91.

### MiRNAs involved in the formation of VM

A large number of studies support a role for miRNAs in influencing tumor cell invasion, proliferation and metastasis by targeting different genes, such as many classical markers of VM. However, the full spectrum of miRNAs activity in regulating the tumor VM remains to be elucidated. The majority of miRNAs regulating the VM process have been identified as VM suppressors, suggesting that this class of molecules could be potential antitumor therapeutics. Hsa-miR-299-5p is involved in the regulation of breast cancer cells, its downregulation increases the expression of osteopontin (OPN), which is a protein secreted by a sub-population of cells, called SFCs, which is required for tumorigenicity and the VM forming ability 92. Other functional studies revealed that both miR-26b and miR-200a are key downregulators of EphA2 in glioma and ovarian cancer cells, suppressing VM and invasion 93, 94. In HCC, miR-1236 downregulates the PTEN/PI3K/AKT pathway by targeting the 3'UTR of AFP mRNA, causing the reduction of VM 95. In human bladder cancer cells, MiR-124 competitively binds to the 3'UTR of UHRF1 mRNA, contributing to the reduction of UHRF1. MiR-124 levels are inversely correlated with the expression of MMP-2, MMP-9, and VEGF, ultimately attenuating cellular migration, invasion, angiogenesis, and VM formation 96. Likewise, in cervical cancer cells, a study showed that miR-124 exerts a negative effect on angiomotin-like protein 1 (AmotL1), which regulates the EMT phenotype, leading to vasculogenic network suppression 97. Additionally, miR186 acts as a Twist1 mediator, dramatically repressing VM formation capacity, EMT, tumorigenesis, and metastasis ability of prostate cancer cells 98. Furthermore, MiR-158-3p plays a suppressive role in VM formation of malignant glioma cells by inhibiting the ROCK1-dependent stress fiber formation 99. Another study elucidated that in breast cancer cells, miR-193b has a downstream effect on dimethylarginine dimethylaminohydrolase 1, which is a newly-discovered mediator of VM 100. In glioma cells, miR-9 and miR-Let-7f were found to be tumor suppressors. MiR-9 was identified due to its negative effect on Stathmin, and leads to a VM-forming failure 101. MiR-Let-7f instead represses periostin expression, directly inhibiting VM formation 102. MiR-27a-3p is considered a key mediator of Twist 1 in HCC cells, where it decreases the expression of VE-cadherin and suppresses EMT signaling, reducing tumor invasion and VM levels 103. Similarly to miR-27a-3p, in ovarian cancer cells, miR-27b decreases angiogenesis and VM formation by binding to the 3'UTR of VE-cadherin mRNA 104. MiR-101 inhibits cancer-associated fibroblast (CAF)-promoted VM in HCC cells through a novel regulatory network, which involves the TGF-β and SDF1-mediated VE-cadherin/MMP-2/LAMC2 signaling pathway 68. A recent study showed that miR-204 in breast cancer cells exerted a positive effect on VM by directly and indirectly regulating the expression levels of 13 proteins involved in multiple signaling pathways including PI3K/AKT, RAF1/MAPK, VEGF, and FAK/SRC 105. Moreover, Yarely et.al demonstrated the role of miR-765 in VM formation in the SKOV3 ovarian cancer cell line, through the modulation of the VEGFA/AKT1/SRC-α axis 106. Additionally, recent findings suggested that in the MDA-MB 231 breast cancer cell line two miRNAs, miR-125a and let-7e, which are highly expressed in ECs, inhibit the activation of IL-6 signaling to suppress VM formation 107 (Table 1).

### LncRNAs involvement in VM formation

LncRNAs were found to serve a similar function to miRNAs in regulating the VM forming process. A recent study showed that in gastric cancer, knockdown of the lncRNA MALAT1 reduced the expression of VE-cadherin, β-catenin, MMP-2, -9, -14, p-ERK, p-FAK, and p-paxillin and impaired VM formation, suggesting that MALAT1 contributes to angiogenesis and VM 108. A further study reported that in NSCLC the MALAT1/miR145-5p/NEDD9 signaling pathway mediated by the estrogen receptor β promoted VM formation and cell invasion 109. The LNC00339 RNA was reported to promote glioma VM formation by targeting the miR-539-5p/TWIST1/MMPs pathway 110. Recent evidence showed that in glioma cells, the HOXA cluster antisense RNA 2 (HOXA-AS2) lncRNA played a negative role in VM formation. Knockdown of HOXA-AS2 in glioma cells upregulates miR-373, which targets EGFR regulating the expression of VE-cadherin, MMP-2, MMP-9, and PI3K/AKT pathway proteins 111. The LNC00312 RNA promoted VM formation in lung adenocarcinoma by directly binding to the transcription factor Y-Box Binding Protein 1 13. Ke et.al reported that in Osteosarcoma, the lncRNA n340532 facilitated VM formation through the TGF-β signaling pathway 112 (Table 1).

### CircRNAs involvement in VM formation

Circular RNAs (circRNAs) are novel RNA molecules with a covalently closed circular structure, which are highly expressed in eukaryotic transcriptomes. A study showed that knockdown of circRNA ZNF292 in HCC resulted in the suppression of cell proliferation and VM formation 113 (Table 1).

An increasing amount of evidence showed that cell viability migration, invasion, and VM formation can be affected by miRNA, lncRNA and circRNA. Thus, novel therapies targeting these three molecules are needed the effective treatment of advanced cancer. Further investigations validating the functions of miRNA, lncRNA and circRNAs in VM formation are necessary.

### VM significance in clinical practice

Routinely assessing the presence of VM is critical for clinical practice. For some malignant cancer biopsies, VM can be diagnosed with IHC staining. The current golden standard for the detection of VM is the positive PAS and negative CD31 staining of vessel-like structures. In a study on non-functioning Pituitary Adenomas (NFPAs), the presence of VM was confirmed by histological staining in 22/49 (44.9%) of the analyzed specimens, but the possible link between VM and NFPAs has not been further investigated 114. Zhang et al. 115 showed that 12 of 17 (70.6%) intracranial hemangiopericytoma samples were VM-positive and associated with tumor recurrence. Other than IHC staining, VM presence in a clinical setting can be detected, using novel molecular imaging technologies, thanks to the availability of contrasting agents that can enter inside the VM tubular structure. A study using the dynamic micro-MRI technique in the WIBC-9 breast cancer xenograft showed a significant blood flow through the tumor, demonstrating the tumor tissue perfusion, which is consistent VM histological features 116. Likewise, Yamamoto et al. used MRI to prove the VM presence in malignant gliomas, describing the radiological features of VM structures 117. Additionally, Doppler imaging of microbeads circulation in human melanoma xenografts showed the physiologic perfusion of blood between the endothelial-lined mouse vasculature and VM networks 11. Furthermore, confocal Indocyanine Green Angiography was used in uveal melanoma to detect the blood circulation in VM patterns 118 (Table 2). A correct VM identification with the help of these techniques is critical for clinical practice and it is necessary for researchers to understand the biologic processes governing VM in living organisms at a cellular and molecular level in living organisms.

Numerous studies showed that VM is closely associated with distant metastasis, a higher recurrence rate, and a shorter survival rate. A study by Lv et al. showed that gastric carcinoma patients with VM have a higher histological grade, more hematogenous metastasis, and a shorter overall and disease-free survival compared to non-VM patients 119. A meta-analysis summarizing the results of 36 clinical studies representing 3609 patients affected by malignant cancers showed that a positive VM status significantly predicted lower overall survival 120. Similarly, another study demonstrated that high grade gliomas had a higher incidence of VM than low grade gliomas, with VM positivity being correlated to a poor prognosis for gliomas patients 121. A meta-analysis including 22 studies representing 2411 patients showed that VM was a poor prognosis factor for digestive cancer patients and positively correlated with tumor differentiation, lymph node metastasis, and TNM stage 122. In addition, Stuart et al. showed that the presence of VM in SCLC specimens decreases tumor latency and negatively affects cisplatin efficacy 4. In contrast, a study reported that VM has no prognostic impact in pT3 and pT4 cutaneous melanomas 123.

### VM and cancer therapeutic

The purpose of tumor angiogenesis is to provide adequate blood supply and nutrition for the cancer cells growth. Therefore, many conventional antiangiogenic drugs that attenuate the EC growth or accelerate EC death are widely used in clinical practice, even if their therapeutic effect is partially limited. One of the causes that could explain the limited efficacy of antiangiogenic drugs is the presence of VM, which is endothelial cells-independent. Furthermore, many antiangiogenic drugs could generate hypoxia due to the blood supply blocking, thus inadvertently contributing to VM formation and tumor proliferation. Thus, anti-VM therapies to treat different tumors type should be considered.

Encouragingly, different studies focusing on targeting VM-related molecules with novel anticancer agents to inhibit VM formation have demonstrated the viability of this strategy. Cilengitide, an inhibitor of αvβ5 integrins, represses VM in aggressive melanoma by reducing the expression of VEGFR-2 and NRP-1 124. Doxycycline have an anti-VM potential in HCC through EMT process inhibition 125. Curcumin restrains VM channel formation in HCC cells by mediating the STAT3 and the PI3K/AKT signaling pathways, and in laryngeal squamous cell carcinoma by mediating the JAK-2/STAT-3 pathway 45, 126. Galunisertib, a TGF-β1 inhibitor, is currently under clinical trials in glioma patients, where it exhibited an inhibitory effect on VM activity by regulating the astrocytes cells, which relies on TGF-β1 secretion, and by decreasing the expression of VE-cadherin and smooth muscle actin-α, reducing the phosphorylation of AKT and FLK 127. Verteporfin, an FDA-approved photosensitizer, that has been clinically used for the treatment of age-related macular degeneration, was recently found to suppress VM in pancreatic ductal adenocarcinoma by inhibiting MMP-2, VE-cadherin, and a-SMA expression 128. Norcantharidin (NICD) suppresses the VM network formation both in human gallbladder carcinoma and in melanoma by downregulating the expression of PI3K, MMP-2, MTI-MMP, and Ln-5γ2 129, 130. Furthermore, a study found that Niclosamide, an oral anti-helminthic drug, has a wide application in oral cancer, where it downregulates the expression of VEGFA, MMP-2, ROCK1, Cdc42, and STAT3 and upregulates the levels of miR-124, ultimately preventing VM formation 131. Additionally, several traditional Chinese medicines also exert an anti-VM effect in various cancer types. A study reported that Hinokitiol, also known as a tropolone-related natural compound, has anti-VM activity in breast cancer cells by decreasing the EGFR protein expression 132. Paris polyphylla was able to block VM in human osteosarcoma, by reducing the expression of FAK, Mig-7, MMP-2, and MMP-9 133. Triptonide is a novel VM inhibitor that in pancreatic cancer cells decreases the expression of VE-cadherin and chemokine ligand 2 (CXCL2) genes 134. Celastrus orbiculatus extract (COE), a mixture of terpenoids, can effectively suppress the angiogenesis and VM formation in HCC cells by suppressing Notch1 and Hes1 expression 135. Luteolin is a flavonoid extracted from green plants that was found to inhibit VM tube formation in gastric cancer through downregulating the Notch1-VEGF signaling pathway 64. Polyphyllin I, isolated from *Rhizoma paridis* saponins, impaired VM formation in HCC cells by blocking the PI3k-Akt-Twist1-VE-cadherin pathway 136. A novel peptide, KVEPQDPSEW, isolated from abalone (Haliotis discus hannai), effectively inhibited VM formation in HT1080 cells by negatively regulating MMPs, VEGF, and AKT/mTOR signaling pathways 137. *In vitro* and *in vivo* experiment revealed that JQ1, a bromodomain and extraterminal domain inhibitor, suppressed VM in pancreatic ductal adenocarcinoma cells by inhibiting the ERK1/2-MMP2/9 signal pathway. SCH772984, an ERK1/2 inhibitor, strongly suppressed VM formation in the PDAC cell line, implying a positive correlation between VM and p-ERK1/2 expression 138. Ethoxy-erianin phosphate (EBTP) is an erianin analog that blocks VM in indoleamine 2,3-dioxygenase -induced Lewis lung cancer cells by regulating the levels of MMP-2, MMP-9, and STAT3 139. In addition, our previous study reported that Incarvine C restrains the vessel-like structure formation in HCC by blocking ROCK expression 140 (Table 3).

## Conclusions

VM is a biological process closely correlated with tumor invasion, migration, and progression. The discovery of VM provides new therapeutic strategies for patients battling with cancer, which are currently limited by treatment with conventional antiangiogenic agents. Hence, the combination of VM inhibitors and anti-angiogenic therapies may be promising therapeutic strategies. This review examined different VM-related markers, including miRNAs, IncRNAs, and circRNAs which can provide to researchers a deeper understanding of the underlying molecular mechanisms. In addition, further studies focusing on the clinical applications of novel agents targeting VM can contribute to the development of more effective therapies.

## Figures and Tables

**Table 1:** MiRNA, lncRNA and CircRNA involved in VM.

	Genes	Targets	Effects on VM	Cancer Type	References
miRNA	Hsa-miR-299-5p	OPN	Promote	Breast cancer	92
	MiR-26b	EphA2	Suppress	Glioma	93
	MiR-200a	EphA2	Suppress	Ovarian cancer	94
	MiR-1236	PTEN/PI3K/AKT	Suppress	HCC	95
	MiR-124	UHRF1, MMP-2, MMP9, VEGF AmotL1	Suppress	Bladder cancer Cervical cancer	96,97
	MiR186	Twist1	Suppress	Prostate cancer	98
	MiR-158-3p	ROCK1	Suppress	Glioma	99
	MiR-193b	DDAH1	Suppress	Breast cancer	100
	MiR-9	STMN1	Suppress	Glioma	101
	MiR-Let-7f	POSTN	Suppress	Glioma	102
	MiR-27a-3p	Twist1, VE-cadherin	Suppress	HCC	103
	MiR-27b	VE-cadherin	Suppress	Ovarian cancer	104
	MiR-101	TGF-β, SDF1 VE-cadherin/MMP2/LAMC2	Suppress	HCC	68
	MiR-204	PI3K/AKT, RAF1/MAPK, VEGF, and FAK/SRC	promote	Breast cancer	105
	MiR-765	VEGFA/AKT1/SRC-α	Suppress	Ovarian cancer	106
	MiR-125a MiRlet-7e	IL-6	Suppress	breast cancer	107
lncRNA	MALAT1	VE-cadherin, β-catenin, MMPs, p-ERK, p-FAK, p-paxillin	Promote	Gastric cancer	108
		miR145-5p/NEDD9	Promote	Non-small cell lung cancer	109
	LNC00339	miR-539-5p/TWIST1/MMPs	Promote	Glioma	110
	HOXA-AS2	miR-373, EGFR VE-cadherin, MMP-2, MMP-9 , PI3K/AKT	Suppress	Glioma	111
	LNC00312	YBX1	Promote	lung adenocarcinoma	13
	lncRNAn340532	TGF-β	Promote	Osteosarcoma	112
CircRNA	cZNF292	hypoxia	Promote	HCC	113

**Table 2:** Identification methods of VM

Identification methods	Cancer Type	References
IHC	Non-functioning Pituitary Adenomas	114
	Intracranial hemangiopericytoma	115
MRI	Breast cancer	116
	Gliomas	117
Doppler imaging	Melanoma	11
Confocal Indocyanine Green Angiography	Uveal Melanoma	118

**Table 3:** Therapeutic agents targeting VM

Therapeutic agents	Molecular targets or function	Cancer Type	References
Cilengitide	αvβ5 integrins,VEGFR-2, NRP-1	Melanoma	124
Doxycycline	EMT inhibition	HCC	125
Curcumin	STAT3, PI3K/AKT	HCC	45
	JAK-2/STAT-3	Laryngeal squamous cell carcinoma	126
Galunisertib	Astrocytes, SMα, Akt,Flk	Glioma	127
Verteporfin	MMP-2,VE-cadherin, a-SMA	Pancreatic ductal adenocarcinoma	128
Norcantharidin	PI3K, MMP-2, MTI-MMP,Ln-5γ2	Gallbladder carcinoma, melanoma	129,130
Niclosamide	VEGFA, MMP-2, ROCK1, Cdc42, STAT3, MiR-124	Oral cancer	131
Hinokitiol	EGFR	Breast cancer	132
Paris polyphylla	FAK, Mig-7, MMP-2,MMP9	Osteosarcoma	133
Triptonide	VE-cadherin, CXCL2	Pancreatic cancer	134
Celastrus orbiculatus extract	Notch1, Hes1	HCC	135
Luteolin	Notch1-VEGF	Gastric cancer	64
Polyphyllin I	PI3k-Akt-Twist1-VE-cadherin	HCC	136
KVEPQDPSEW	MMPs , VEGF AKT/mTOR	Fibrosarcoma	137
JQ1	ERK1/2-MMP2/9	Pancreatic ductal adenocarcinoma	138
SCH772984	ERK1/2	Pancreatic ductal adenocarcinoma	138
Ethoxy‐erianin phosphate	MMP‐2, MMP‐9, and STAT3	2,3‐dioxygenase -induced Lewis lung cancer	139
Incarvine C	ROCK	HCC	140

## Abbreviations

VM: Vasculogenic mimicry
ECs: endothelial cells
miRNAs: microRNAs
LncRNAs: long non-coding RNAs
HCC: hepatocellular carcinoma
MVs: mosaic vessels
EVs: endothelium-dependent vessels
EMT: epithelial-to-mesenchymal Transition
CSCs: cancer stem cells
ALDH1: Aldehyde dehydrogenase 1
ZEB1: Zinc finger E-box binding homeobox 1
MMP: Matrix metalloproteinase
EGFR: epidermal growth factor receptor
VE-cadherin: Vascular endothelial-cadherin
EphA2: Eph receptor tyrosine kinase A2
SCLC: small cell lung cancer
FAK: focal adhesion kinase
PI3K: Phosphoinositide 3-Kinase
ERK: extracellular signal-regulated kinase
MEK: mitogen extracellular kinase
CDK-5: cyclin-dependent kinase 5
VEGF-A: Vascular endothelial growth factor-A
VEGFR1: vascular endothelial growth factor receptor 1
SEMA4D: semaphorin4D
PEDF: pigment epithelium-derived factor
TF: Tissue factor
TFPI: TF pathway inhibitor
TGF-β: transforming growth factor β
NICD: Notch intracellular domain
CAF: Cancer-associated fibroblast
HIF-1: hypoxia-inducible factor-1
MIF: migration inhibitory factor
CXCR4: C-X-C motif chemokine receptor 4
COX-2: Cyclooxygenases-2
PGE2: prostaglandin E2
PKC: protein kinase C
ROCKs: Rho kinases
MLC: myosin light chain
OPN: osteopontin
AmotL1: angiomotin-like protein 1
DDAH1: dimethylarginine dimethylaminohydrolase 1
STMN1: Stathmin1
POSTN: periostin
ERβ: estrogen receptor β
YBX1: Y-Box Binding Protein 1
NICD: Norcantharidin
CXCL2: chemokine ligand 2
COE: Celastrus orbiculatus extract
PPI: Polyphyllin I
AATP: KVEPQDPSEW
BET: bromodomain and extraterminal domain
PDAC: pancreatic ductal adenocarcinoma
EBTP: Ethoxy‐erianin phosphate
